# A Novel Diagnostic Model for Primary Adrenal Lymphoma

**DOI:** 10.3389/fendo.2021.636658

**Published:** 2021-04-02

**Authors:** Kai Yu, Qingping Xue, Fangli Zhou, Haoming Tian, Qiao Xiang, Tao Chen, Yan Ren

**Affiliations:** ^1^ Adrenal Center, Department of Endocrinology and Metabolism, West China Hospital, Sichuan University, Chengdu, China; ^2^ Department of Public Health, Chengdu Medical College, Chengdu, China; ^3^ West China School of Public Health, West China Fourth Hospital, Sichuan University, Chengdu, China

**Keywords:** primary adrenal lymphoma, nonfunctional adrenal mass, pheochromocytoma, diagnosis, nomogram

## Abstract

**Objective:**

Primary adrenal lymphoma (PAL) is easily misdiagnosed as other adrenal masses, such as adrenocortical carcinoma and pheochromocytoma, but patients with PAL benefit little from surgery. The diagnostic method for PAL thus far is limited to adrenal biopsy. In our study, we aimed to develop a quick and efficient diagnostic method for PAL.

**Methods and Results:**

At the same institution, 505 patients (between 2009 and 2019) and 171 patients (between 2019 and 2020) were separately included in the primary and validation studies. Univariate and multivariate analyses were conducted to evaluate clinical manifestations, laboratory findings, and radiological characteristics. Four determinants (age, bilateral masses, high-density lipoprotein cholesterol, and lactate dehydrogenase) were selected and further incorporated into a regression model to screen PAL. Accordingly, the nomogram was developed for clinical practice. In the primary study, the nomogram showed good discrimination, with an area under the receiver operating characteristic (ROC) curve (AUC) of 95.4% (95% CI, 90.6%–100.0%). Further validation study verified the efficacy of the nomogram, with an AUC of 99.0% (95% CI, 96.9%–100.00%) and 100.0% in all patients and patients with bilateral masses, respectively, and a sensitivity/specificity/positive predictive value (PPV)/negative predictive value (NPV) of 66.67%/99.40%/66.67%/99.40%, 66.67%/100%/100%/92.86%, 50%/99.20%/50%/99.20%, and 100%/100%/100%/100%, in all patients, patients with bilateral adrenal masses, patients with nonfunctional adrenal masses, and patients with positive catecholamine results, respectively. The validation study also revealed a diagnostic specificity of 99.35% and 100% for patients with a unilateral adrenal mass and functional PCC, respectively.

**Conclusions:**

The presented nomogram is the first user-friendly diagnostic model for PAL that simplifies the complex diagnostic process into personalized numeric estimates. We deem that patients who score below 50 are less likely to have PAL. We suggest that clinicians should arrange adrenal biopsy and surgery for patients with nonfunctional tumors and overt catecholamine-secreting tumors, respectively, who receive a score of 50 points or higher to confirm the diagnosis as soon as possible.

## Introduction

Primary adrenal lymphoma (PAL) is a rare adrenal malignancy constituting 1% of non-Hodgkin’s lymphoma (NHL), and its most common histological type is diffuse large B cell lymphoma (DLBCL) (1, 2). PAL refers to histopathologically confirmed adrenal lymphoma with no previously diagnosed lymphoma at other sites or with coinstantaneous less-predominant lesions at sites other than the adrenal glands (3, 4). With no past history of lymphoma, PAL is more difficult to diagnose. The overall survival (OS) rate for PAL patients is 19.17% at 5 years and drops to 3.33% at 10 years (1, 5). Disappointingly, the manifestations of PAL are not discriminating, leaving a diagnostic challenge for patients, endocrinologists and surgeons to distinguish PAL from other adrenal masses (6).

Approximately 4% of the general population has adrenal masses (7). Though only 5-10% of them are malignant, the prognoses of these adrenal malignancies are dismal (8). Furthermore, among these adrenal malignancies, the therapeutic regimen for PAL is different (4, 5, 9). Therefore, early diagnosis of PAL is essential. To date, hormone-secreting adrenal lesions, such as adrenal Cushing’s syndrome (ACS), aldosterone-producing adrenocortical adenoma (APA), and aldosterone-producing adrenocortical carcinoma (APC), can be easily screened by hormonal measurements and functional tests. However, it has been reported that PAL and pheochromocytoma (PCC) might simultaneously develop in the same adrenal gland (10). Some PALs can mimic PCC, presenting with moderate to clear elevation of catecholamines (11, 12). PCC preoperative preparation is time-consuming, but the progression of PAL can be extremely aggressive, and patients may even die before receiving chemotherapy (13). Moreover, the diagnosis of hormonally inactive diseases, such as PAL, adrenal metastasis, or silent adrenocortical carcinoma, remains elusive. However, some studies have indicated that nonenhanced computed tomography (CT) has good sensitivity in distinguishing malignant adrenal lesions from benign lesions. Adrenal masses that are smaller than 4 cm and have a density less than 10 Hounsfield units (HUs) are unlikely to be malignant (14). The density of PAL lesions can be variable, and the diameter of PAL ranges from one centimeter to over 10 cm (15–17). In addition, few studies have focused on the differentiation of malignant tumors. In such cases, adrenal biopsy or fine needle aspiration (FNA) may be a diagnostic option when PCC can be fully excluded (14). Therefore, to diagnose PAL, it is essential to screen for PAL in patients with positive catecholamine results and to distinguish patients with PAL from patients with other nonfunctional adrenal masses.

To our knowledge, though not discriminating, PAL is an entity that mainly affects elderly males and both adrenal glands (2). Patients with PAL usually experience fever, abdominal or lumbar pain, weight loss, and adrenal insufficiency (AI) (18). Elevated serum lactate dehydrogenase (LDH) and immune deficiency are common in PAL patients (2). Peripheral lymphocytopenia and a reduction in high-density lipoprotein cholesterol (HDL-C) levels have been reported to be frequent in NHL (19, 20). Additionally, absolute monocyte count was shown to be an independent determinant for central nervous system relapse in patients with DLBCL (21). Nevertheless, few studies have focused on the diagnostic utilities of these variables alone or in combination for PAL.

Thus, in this study, we retrospectively reviewed the medical records of two independent cohorts of patients with nonfunctional adrenal masses and functional PCC from the same institution and aimed to establish a novel diagnostic model for PAL based on clinical, laboratory and radiological data.

## Methods

### Patients and Diagnoses

To establish a new diagnostic model for PAL in hormonally inactive adrenal masses and functional PCC, we retrospectively reviewed a cohort of patients who underwent surgery or adrenal biopsy and had hormonal test results between January 1, 2009, and June 30, 2019 (the primary cohort). This study was approved by the Ethics Committee of our hospital (No. 2019-556). All subjects with nonfunctional adrenal masses and patients who had functional PCC were included in our study. Patients with functional adrenal masses such as ACS, APA and APC that can be easily diagnosed by hormonal examination were excluded.

Diagnoses were confirmed by two experienced physicians independently by reviewing clinical, laboratory, and radiological files from electronic medical records. All adrenal masses were defined as nonfunctional when 1) urine or serum free cortisol and adrenocorticotropic hormone were in the normal reference range, the cortisol circadian rhythm was normal, or cortisol was suppressed in the small-dose dexamethasone suppression test; 2) the ratio of plasma aldosterone to renin activity or the aldosterone level did not meet the primary aldosteronoma definition according to the diagnostic criteria; and 3) urine or serum epinephrine or norepinephrine was less than 3 times the upper limit of the reference range (22–24). In line with former studies, PAL was considered when lymphoma was confined chiefly or wholly to the adrenal glands and proved by histology but with no lymphoma past history (4). Adrenal adenomas, adrenal metastatic tumors, adrenocortical carcinomas, etc. were all diagnosed by histology.

In addition, from July 1, 2019, to June 30, 2020, independent cohorts of patients with adrenal masses based on the same inclusion and exclusion criteria were extracted from the same institution as a validation cohort.

### Variables

Demographical predictors, including age and sex, and clinical risk factors derived from medical records, including abdominal pain, lumbar pain, B symptoms (simultaneous fever, night sweats and weight loss), bilateral masses, immune abnormalities (including autoimmune disease and cancer), hyperglycemia, maximum diameter (D-max), absolute monocyte count, absolute lymphocyte count, HDL-C and LDH, were included in our study. Considering that only four (1 PAL, 1 ACC, 2 PCCs) out of 509 patients in the primary cohort were missing necessary information, those patients were directly deleted without applying multiple imputations. Abdominal pain, lumbar pain, B symptoms, bilateral masses, and immune abnormalities were dichotomized into yes and no, respectively. Hyperglycemia (including diabetes mellitus and prediabetes) was confirmed according to the criteria in the guidelines of the American Diabetes Association or based on prior diagnoses and classified into two groups (yes and no) (25).

### Statistical Analysis

Categorical variables were summarized as frequencies and percentages, while continuous variables were summarized as the mean and standard deviation (SD) if normally distributed or the median and interquartile range (IQR) otherwise. Student’s t-test (for continuous variables), Mann-Whitney U tests (for continuous variables with skewness distributions) and the chi-square test (for categorical variables) were utilized to compare the differences in basic characteristics between cases (29 patients with PALs) and controls (including 15 nonfunctional adenomas, 26 adrenal metastatic tumors, 2 silent adrenocortical carcinomas, 6 cases of adrenal tuberculosis, 2 cases of adrenal hyperplasia, and 425 PCCs) in the primary cohort (26–28). Univariable and multivariable logistic regressions were conducted to evaluate the value of variables that heralded PAL. Possible predictors were screened by univariable logistic regression analysis and multivariable logistic regression, with statistical significance at *P* < 0.05 and with a 95% confidence interval (CI) around their odds ratio (OR) not containing 1.00 (29).

Then, based on a multivariable regression model, a visually predictive nomogram was established using the predictors, and its performance was assessed by discrimination and calibration (30). Briefly, the area under the receiver operating characteristic (ROC) curve (AUC) and its 95% CI were calculated to assess the discriminative performance of the nomogram, and a relatively good discrimination was defined as AUC > 0.75 (31). Then, to evaluate concordance between the observed outcome probabilities and predicted outcome probabilities, a calibration curve derived from regression analysis coupled with the Hosmer–Lemeshow (HL) test was performed (32). P > 0.05 was considered well calibrated. Moreover, bootstraps with 1000 resamples were used for internal validation and plotting calibration curves (33). Then, the nomogram built from the primary cohort was further validated in the validation cohort. Briefly, the validation cohort was further divided into four groups: the unilateral group, bilateral group, nonfunctional group, and functional group. Next, the total points for each patient in the validation cohort were calculated according to the nomogram, and then logistic regression of this cohort in all groups, in the bilateral group, and in the nonfunctional group was performed using total points as a separate factor. Then, the prediction efficacies were evaluated in the validation cohort in all patients and in each group. The performance of the model was measured on the validation cohort by discrimination and calibration using the same methods described above. Finally, comparisons between the nomogram and other indicators were performed with the test.roc function in the pROC package (version 1.16.2) by default (34).

All statistical analyses were conducted by using R version 4.0.1 (R Foundation for Statistical Computing, Vienna, Austria), and *P* < 0.05 was defined as statistically significant.

## Results

### Characteristics of Patients

Between 2009 and 2019, 505 patients were included in the primary cohort, of whom 5.74% had PAL, 54.64% were female, and 22.57% had hyperglycemia. The mean age of the primary cohort was 47.01 years (SD, 14.68 years), the median absolute lymphocyte count was 1.48 10^9^/L (IQR, 1.10-1.88 (10^9/L)), the median level of HDL-C was 1.42 mmol/L (IQR, 1.16-1.81 (mmol/L)) and median LDH level was 164.00 IU/L (IQR, 144.00-199.00 (IU/L)).

**Figure 1:**
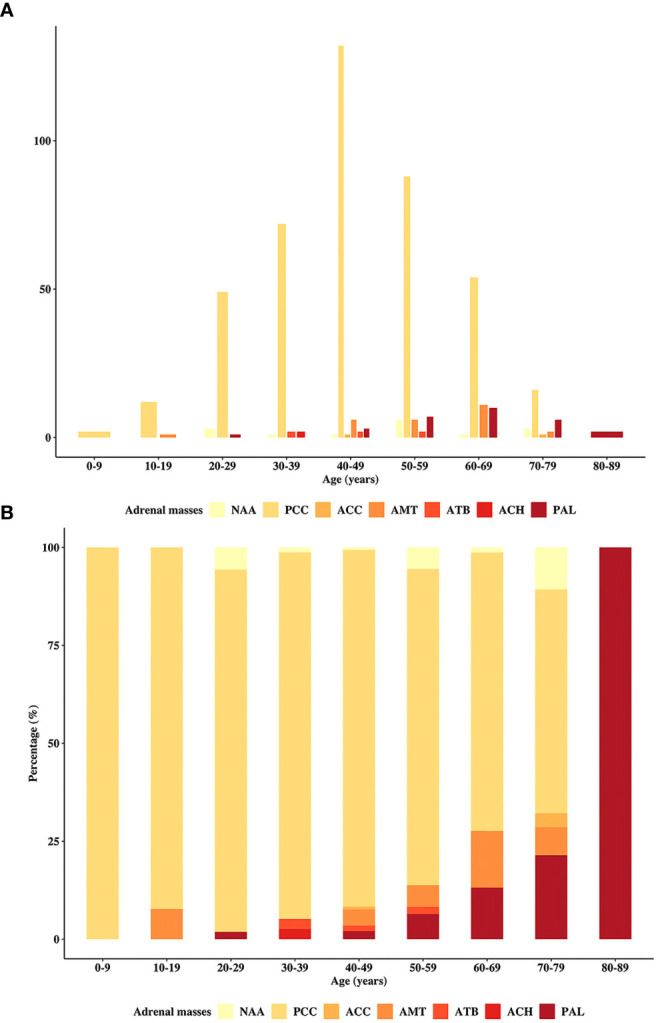
Distribution of the patients in the primary study by age. The incidence of the cohort patients stratified by the age at diagnosis, including **(A)** absolute and **(B)** proportional cases. PAL, primary adrenal lymphoma; NAA, nonfunctional adrenal adenoma; PCC, pheochromocytoma; ACC, adrenocortical carcinoma; AMT, adrenal metastasis; ATB, adrenal tuberculosis; ACH, adrenocortical hyperplasia.

The incidence of individual adrenal disease differed with patient age. **Figure 1** depicts the incidence of each type of adrenal disease grouped by age at diagnosis. **Figures 1A, B** illustrate the absolute incidence and proportional incidence, respectively. The results showed that PCCs remained predominant until the age of 80 years. Adrenal metastatic tumor was the second most common adrenal mass in patients aged 40 to 49 years and 60 to 69 years. In patients aged 50 to 59 years and 70 to 79 years, PAL became the second most predominant. Only two patients in the cohort were over 80 years old, and they both had PAL.

Bilateral adrenal masses accounted for 11.68% and 9.36% of all masses in the primary cohort and validation cohort, respectively (**Table 1**). The etiological distribution of bilateral adrenal disease differed from that of unilateral adrenal disease. In our study, only patients with PAL or adrenal tuberculosis (ATB) were prone to develop bilateral lesions. More patients with PCC, adrenocortical adenoma and adrenal metastasis had unilateral lesions.

**Table 1:** The etiology distribution of patients in the primary and validation cohorts.

Type	The primary cohort (n = 505)		The validation cohort (n = 171)	
	BL (n =59)	UL (n = 446)	BL (n = 16)	UL (n = 155)
PAL	16 (27.12%)	14 (3.14%)	3 (18.75%)	–
NAA	2 (3.39%)	12 (2.69%)	5 (31.25%)	38 (24.52%)
PCC	31 (52.54%)	394 (88.34%)	2 (12.50%)	55 (35.48%)
Functional	26 (44.07%)	307 (68.83%)	2 (12.50%)	41 (26.45%)
Nonfunctional	5 (8.47%)	87 (21.53%)	–	14 (9.03%)
ACC	–	2 (0.45%)	–	4 (2.58%)
AMT	5 (8.47%)	21 (4.71%)	1 (6.25%)	14 (9.03%)
ATB	5 (8.47%)	1 (0.22%)	1 (6.25%)	–
ACH	–	2 (0.45%)	1 (6.25%)	7 (4.52%)
AML	–	–	–	1 (0.65%)
AC	–	–	–	4 (2.58%)
Sarcoma	–	–	–	4 (2.58%)
ML	–	–	2 (12.50%)	19 (12.26%)
NB	–	–	–	2 (1.29%)
GNB	–	–	–	1 (0.65%)
LA	–	–	–	3 (1.94%
GN	–	–	1 (6.25%)	2 (1.29%)
Teratoma	–	–	–	1 (0.65%)
Angioma	–	–	–	–

PAL, primary adrenal lymphoma; NAA, nonfunctional adrenal adenoma; PCC, pheochromocytoma; ACC, adrenocortical carcinoma; AMT, adrenal metastasis; ATB, adrenal tuberculosis; ACH, adrenocortical hyperplasia; AML, angioleiomyolipoma; AC, adrenal cyst; ML, myelolipoma; NB, neuroblastoma; GNB, ganglioneuroblastoma; LA, lymphangioma; GN, ganglioneuroma.

In the primary cohort, compared with controls, patients with PAL were older (*P* < 0.001); had a larger D-max (*P* < 0.001), elevated LDH levels (*P* < 0.001), lower absolute lymphocyte count (*P* < 0.001), and decreased HDL-C levels (*P* < 0.001); were more likely to be male (*P* = 0.015); had lumbar pain (*P* = 0.002); and had bilateral masses (*P* < 0.001) (**Table 2**). In addition, 4 out of 29 patients with PAL had overtly elevated catecholamines preceding surgery.

**Table 2:** The basic characteristics of the primary and validation cohort patients.

	The primary cohort (n = 505)		*P* value	The validation cohort (n = 171)		*P* value
	PAL (n = 29)	non-PAL (n = 476)		PAL (n = 3)	non-PAL (n = 168)	
**Age**	61.55 (13.22)	46.13 (14.31)	<0.001	72.33 (8.02)	48.10 (15.95)	0.028
**Sex:**			0.015			0.116
Female	9 (31.03%)	267 (56.09%)		3 (100.00%)	81 (48.21%)	
Male	20 (68.97%)	209 (43.91%)		0 (0.00%)	87 (51.79%)	
**Immune disorder:**			0.156			1.000
No	28 (96.55%)	413 (86.76%)		3 (100.00%)	136 (80.95%)	
Yes	1 (3.45%)	63 (13.24%)		0 (0.00%)	32 (19.05%)	
**Hyperglycemia:**			0.349			1.000
No	25 (86.21%)	366 (76.89%)		3 (100.00%)	150 (89.29%)	
Yes	4 (13.79%)	110 (23.11%)		0 (0.00%)	18 (10.71%)	
**Abdominal pain:**			0.111			0.327
No	20 (68.97%)	393 (82.56%)		2 (66.67%)	148 (88.10%)	
Yes	9 (31.03%)	83 (17.44%)		1 (33.33%)	20 (11.90%)	
**Lumbar pain:**			0.002			0.044
No	17 (58.62%)	395 (82.98%)		1 (33.33%)	148 (88.10%)	
Yes	12 (41.38%)	81 (17.02%)		2 (66.67%)	20 (11.90%)	
**B symptoms:**			0.057			0.018
No	28 (96.55%)	476 (100.00%)		2 (66.67%)	168 (100.00%)	
Yes	1 (3.45%)	0 (0.00%)		1 (33.33%)	0 (0.00%)	
**Bilateral masses:**			<0.001			0.001
No	13 (44.83%)	433 (90.97%)		0 (0.00%)	155 (92.26%)	
Yes	16 (55.17%)	43 (9.03%)		3 (100.00%)	13 (7.74%)	
**D_max**	7.00 [5.80;10.00]	4.90 [3.58;6.60]	<0.001	10.20 [9.60;10.30]	3.90 [2.40;5.82]	0.006
**M**	0.40 [0.36;0.50]	0.34 [0.26;0.45]	0.018	0.69 [0.62;0.73]	0.41 [0.32;0.52]	0.017
**L**	0.83 [0.53;1.26]	1.52 [1.15;1.90]	<0.001	0.81 [0.80;0.94]	1.54 [1.23;1.87]	0.012
**HDL-C**	0.89 [0.62;1.07]	1.46 [1.19;1.83]	<0.001	0.64 [0.60;1.00]	1.19 [0.95;1.44]	0.111
**LDH**	372.00 [265.00;614.00]	162.00 [142.75;191.25]	<0.001	562.00 [403.50;682.00]	154.00 [133.75;182.50]	0.005

D-max, maximum diameter; M, monocyte; L, lymphocyte; HDL-C, high-density lipoprotein cholesterol; LDH, serum lactate dehydrogenase.

P values were estimated by Student’s t-test or Mann-Whitney U tests for continuous variables and chi-square tests for categorical variables.

For the validation cohort, we studied 171 patients, including 3 with PALs and 168 with other adrenal masses (42 nonfunctional adenomas, 57 PCCs, 4 adrenocortical carcinomas, 15 adrenal metastases, 1 case of ATB, 8 cases of hyperplasia, 1 angioleiomyolipoma, 4 cysts, 21 myelolipomas, 2 neuroblastomas, 1 ganglioneuroblastoma, 2 sarcomas, 1 schwannoma, 3 lymphogiomas, 3 ganglioneuromas, 1 teratoma, 1 eosinophil tumor, and 1 INI-1 deletion myoepithelial carcinoma). Fourteen of the 57 PCCs were functional PCCs. The mean age of the three PAL patients was 72.33 years (SD, 8.02 years), all of them were female and had bilateral lesions, and none of them had glycemic disorders or immune abnormalities. One of them suffered from lumbar pain and had B symptoms. One had both lumbar and abdominal pain, and 1 was asymptomatic. The median maximal diameter of the three PAL patients’ lesions was 10.20 cm (IQR, 9.60-10.30 (cm)). They had a larger D-max (*P* = 0.006), higher monocyte count (*P* = 0.017), lower lymphocyte count (*P* = 0.012), and higher LDH level (*P* = 0.005). These PAL patients had lower median HDL-C levels, but the difference was not statistically significant (**Table 2**). Among the 3 PAL patients, one had a borderline positive metanephrine result.

### Determinant Factors for PAL

The results of the univariable and multivariable logistic regressions are summarized in **Table 3**. Univariable logistic regression showed that age, sex, lumbar pain, bilateral masses, D-max, absolute lymphocyte count, HDL-C and LDH were significantly associated with PAL. Furthermore, age (OR, 1.07, 95% CI, 1.02-1.14), bilateral masses (OR, 7.37, 95% CI, 1.44-39.29), HDL-C (OR, 0.12 95% CI, 0.01-0.68), and LDH (OR, 1.01, 95% CI, 1.01-1.02) were verified as independent determinants for PAL in multivariable logistic regression (**Table 3**).

**Table 3:** Univariable and multivariable logistic regression analysis results for the nomogram.

Characteristics		Univariate analysis		Multivariate analysis	
		OR (95% CI)	*P* value	OR (95% CI)	*P* value
**Age**		1.09 (1.05, 1.13)	<0.001	1.07 (1.02, 1.14)	0.002
**Sex**	Female	1.00 (ref.)	0.011	1.00 (ref.)	0.567
	Male	2.84 (1.27, 6.36)		0.68 (0.15, 3.02)	
**Lumbar pain**	No	1.00 (ref.)	0.002	1.00 (ref.)	0.124
	Yes	3.44 (1.58, 7.48)		2.93 (0.62, 14.76)	
**Bilateral masses**	No	1.00 (ref.)	<0.001	1.00 (ref.)	0.004
	Yes	12.39 (5.59, 27.48)		7.37 (1.44, 39.29)	
**D-max**		1.30 (1.16, 1.45)	<0.001	0.96 (0.72, 1.24)	0.710
**L**		0.07 (0.03, 0.19)	<0.001	0.41 (0.10, 1.31)	0.116
**HDL-C**		0.02 (0.01, 0.06)	<0.001	0.12 (0.01, 0.68)	0.007
**LDH**		1.01 (1.01, 1.02)	<0.001	1.01 (1.01, 1.02)	<0.001

D-max, maximum diameter; L, lymphocyte count; HDL-C, high-density lipoprotein cholesterol; LDH, serum lactate dehydrogenase.

### The Nomogram for PAL

**Figure 2:**
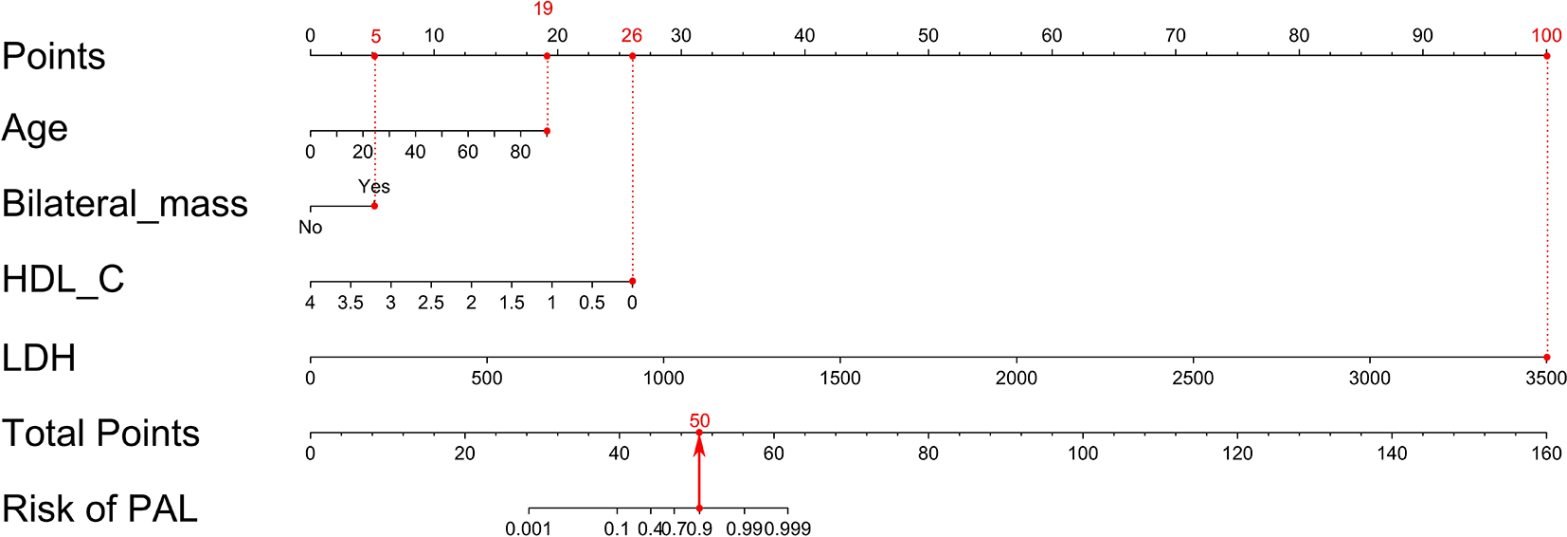
Nomogram to predict PAL based on age, bilateral masses, HDL-C and LDH. To build the nomogram, the first step was to locate each statistically significant variable (including age, bilateral masses, HDL-C and LDH) on the relevant axis, and then a straight line was drawn upward to the point axis on the top to gain the points for each predictor. The second step was to sum all points gained from each predictor to calculate the total points and locate them on the total point axis. Finally, a straight line was drawn at the bottom to indicate the probability of developing PAL. HDL-C, high-density lipoprotein cholesterol; LDH, serum lactate dehydrogenase; PAL, primary adrenal lymphoma.

**Figure 3:**
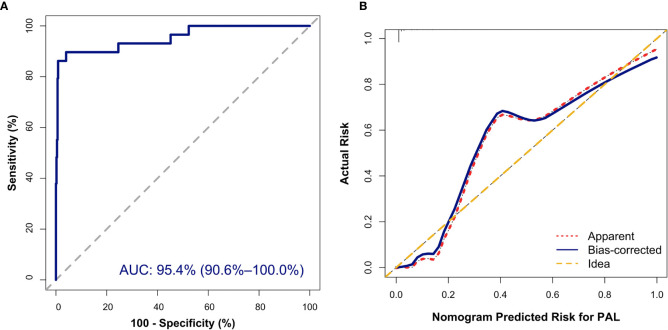
Internal validation of the nomogram to diagnose PAL. **(A)** Discrimination: The area under the receiver operating characteristic (ROC) curve (AUC) was 95.4% (95% CI, 90.6%–100.0%). **(B)** Calibration curve of the nomogram to depict agreement between predicted risks and actual outcomes of PAL. The horizontal axis represents the predicted risk of PAL, and the vertical axis represents the actual risk of the tumor. The 45° dashed line indicates perfect prediction by an ideal model. The dotted and solid lines indicate the observed (apparent) nomogram performance before and after bootstrapping.

The nomogram was built on the basis of multivariable logistic regression analysis as follows: LogitP = -7.825450 + 0.087609 * Age + 2.129424 * (Bilateral masses = 1) - 2.683870 * HDL-C + 0.011778 * LDH, as shown in **Figure 2**. The AUC of the ROC curve for the PAL screen was 95.4% (95% CI, 90.60%–100.00%), indicating good discrimination (**Figure 3A**).

**Figure 4:**
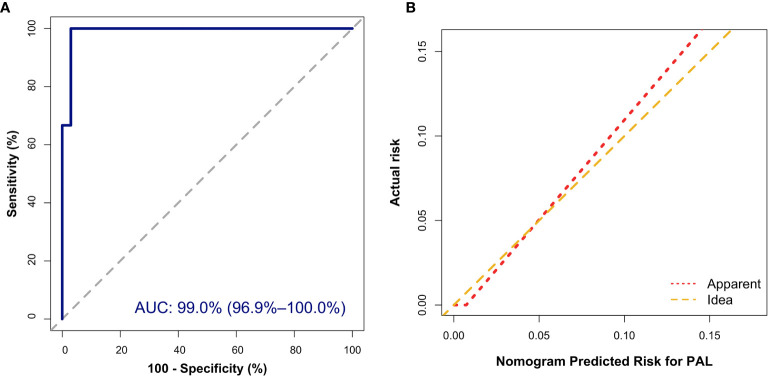
External validation of the nomogram to diagnose PAL. **(A)** Discrimination: The area under the receiver operating characteristic (ROC) curve (AUC) was 99.0% (95% CI, 96.9%–100.0%). **(B)** Calibration curve of the nomogram to depict agreement between predicted risks and actual outcomes of PAL. The horizontal axis represents the predicted risk of PAL, and the vertical axis represents the actual risk of the tumor. The 45° dashed line indicates perfect prediction by an ideal model, and the solid line indicates the observed (apparent) nomogram performance.

In addition, the Hosmer–Lemeshow (HL) test showed no statistical significance (*P* = 0.305), meaning a good fitting of this model. Furthermore, the calibration plot also indicated good concordance between the nomogram-predicted probabilities and actual probabilities of identifying PAL (**Figure 3B**). In the validation cohort, the ROC curve indicated that the model had good discrimination in all patients (**Figure 4A**) and in patients with bilateral masses; the AUCs of the nomogram were 99.0% (95% CI, 96.9%–100.00%) and 100%, respectively. No statistical significance was found in the Hosmer–Lemeshow (HL) test (*P* = 0.604, *P* = 1.000), indicating a goodness of fit of this model. The predicted probabilities and actual probabilities for PAL in all validation cohorts agreed well in the calibration plots (**Figure 4B**).

According to the nomogram, patients who scored over 50 points had a greater than 90% possibility of having PAL. When the algorithm was applied to the validation cohort, one PAL patient was misdiagnosed as non-PAL, and 1 non-PAL patient with a unilateral adrenal lesion was misdiagnosed as PAL; 1 PAL patient with a nonfunctional adrenal mass was misdiagnosed as non-PAL, and 1 non-PAL patient with a nonfunctional adrenal mass was misdiagnosed as PAL. The predictive efficacy of the nomogram in patients with unilateral disease, bilateral disease, nonfunctional disease, and functional disease is shown in **Table 4**. Therefore, our nomogram has ideal specificity in patients with unilateral adrenal lesions, bilateral adrenal lesions, nonfunctional adrenal lesions, functional lesions, and adrenal lesions.

**Table 4:** The predictive efficacy of the nomogram in validation cohort patient groups.

	Sensitivity	Specificity	PPV	NPV
All	66.67%	99.40%	66.67%	99.40%
Bilateral group	66.67%	100.00%	100%	92.86%
Unilateral group	UK	99.35%	UK	UK
Nonfunctional group	50%	99.2%	50%	99.2%
Functional group	100%	100%	100%	100%

PPV, positive predictive value; NPV, negative predictive value; UK, unknown.

### Comparison of Predictive Accuracy Between the Nomogram and a Single Independent Factor

As shown in **Table 3**, the hazard ratio of HDL-C was higher than the hazard ratios for other variables. The PAL diagnostic powers of the nomogram and HDL-C level were compared. The AUC for the diagnosis of PAL based on the HDL-C level was 87.9% (95% CI, 81.2%–94.6%), which was significantly lower than the AUC for the diagnosis of PAL based on the nomogram (95.4%; P < 0.001).

## Discussion

PAL is an extremely aggressive adrenal malignancy. Early diagnosis of PAL is essential, but no quick diagnostic method is available. Patients with PAL might die before pathological confirmation (13, 35). In addition, recent studies suggested that unlike other adrenal malignancies, adrenalectomy preceding surgery provided no benefit to PAL patients (4, 5, 9). In contrast, surgery may expose PAL patients to the risk of delay in starting chemotherapy (36). It is recommended that adrenal biopsy should be performed only if the expected findings are likely to alter the management of the individual patients and PCC has been excluded (14). Adrenal masses are detected in approximately 4%-10% of patients receiving cross-sectional imaging (37, 38). The questions are, among patients with adrenal masses, who may have PAL and who should undergo an adrenal biopsy? Imaging studies, such as CT and magnetic resonance imaging (MRI), are capable of revealing adrenal malignancies; however, further diagnosis of PAL is impossible (14). Furthermore, some PAL lesions can be small and hypodense, overlapping with the imaging features of benign disease (17, 36). In addition, up to 40% of adrenal adenomas have an unenhanced attenuation level over 10 (39). This study was the first to develop an individualized and user-friendly model for the diagnosis of PAL and the management of adrenal incidentaloma.

In our study, compared with patients suffering from other adrenal diseases, patients with PAL were more likely to be elderly males; to experience lumbar pain; to have bilateral lesions; to experience B symptoms; and to have a larger D-max, higher LDH levels, lower monocyte counts, lower lymphocyte counts and lower HDL-C levels (**Table 2**). More importantly, after adjusting the interaction effects, our results showed that older age, bilateral masses, lower HDL-C level and higher LDH level were independently associated with PAL discrimination. However, in the study conducted by Lomte et al., age and bilateral proclivity showed poor discrimination because of their overlaps with PAL, adrenal metastasis and pheochromocytoma (40). Indeed, bilateral adrenal incidentaloma affects 0.3-0.6% of the general population (41). The etiologies of bilateral adrenal incidentaloma are diverse. These studies indicated that a single indicator might not be strong enough to screen PAL. Ozimek et al. reported that LDH levels increased in both PAL and ACC, but they concluded opposite results to our study (16). As their result was merely derived from a comparison between 2 patients, one with PAL and another with ACC, we found that their findings were not reliable. A case-control study unveiled the reverse association of statins, HDL-C stimulators, and NHL (OR 0.61, 95% CI 0.45-0.84) (42). Recently, studies further validated that NHL was an inflammation-induced lymphoma with low circulating HDL-C (42, 43). More specifically, HDL-C is capable of inhibiting the cytokine-triggered endothelial cell adhesion and blocking the activity of lymphocytes and monocytes, thus attenuating the inflammatory response (44). The adrenal gland has a different histological behavior than nodal or extra-adrenal lymphomas in that the adrenal gland does not have lymphatic tissue (2). The pathogenesis of PAL is not yet well understood. No study has investigated the HDL-C level of PAL patients. Derived from multivariate regression analysis, our study showed that a lower HDL-C level was the strongest determinant of PAL. We inferred that HDL-C might also have an important role in the lymphomagenesis of the adrenal gland.

The real-world data of individual patients can be sophisticated. The use of a single index has many drawbacks. Briefly, a significant age difference was observed between patients with PAL and patients with other adrenal diseases, and the age span of patients with PAL and that of patients with other adrenal diseases overlapped (40). Patients of the same age might have different adrenal problems. Individual indices linked PAL diagnosis only to descriptive variables, not to determinant variables. Our study showed that HDL-C was the most powerful indicator, but with much lower discrimination efficiency (compared with that of the nomogram, P < 0.001). Namely, patients with lower HDL-C levels were merely more likely to have PAL without incorporating other variables, such as bilateral proclivity. Given the limitation of the simple index, the nomogram can incorporate variables into an individualized, simple, numeric estimate (30). We established a nomogram to assess the possibility of PAL combined with demographic, radiological and laboratory findings, with an AUC of 95.4%.

Our study showed that 11.09% of the included patients (11.68% in the primary study and 9.36% in the validation study) had bilateral adrenal masses. These findings were slightly inferior to those of previous studies (41, 45). The results of some studies suggested differences in patients with bilateral adrenal disease and unilateral adrenal disease (45). In agreement with former studies, our data showed that patients with PCC and NAA were prone to unilateral lesions, while more patients with PAL or ATB had bilateral disease. However, we found that unilateral adrenal metastasis was more frequent. Further validation tests verified the efficacy of the nomogram, with an AUC of 99.0%. For nonfunctional patients who score over 50 points, direct surgery should be avoided, and an adrenal biopsy should be arranged as soon as possible. Moreover, our findings indicated that the specificity of the nomogram was not significantly weakened by different disease distributions between the unilateral and bilateral groups, and the predictive specificity in patients with unilateral lesions, bilateral lesions, and both was 99.4%, 100%, and 99.35%, respectively. That is, patients who score less than 50 points are less likely to have PAL. In this condition, unilateral adrenalectomy can be considered for unilateral adrenal incidentaloma with suspicious malignant features on imaging, as well as certain tumors larger than 4-6 cm (14). Other causes, such as tuberculosis, should be taken into consideration and carefully evaluated. Similar to the case reported by Cao et al., the 4 patients who were highly suspected of having PCCs in our primary study and the undiagnosed patient in the validation study were ultimately revealed to have PALs based on postoperative pathology (12). According to the nomogram, patients in our validation study scored over 50. Therefore, we want to warn that physicians and surgeons should be cautious about PAL when the score of catecholamine-elevated patients exceeds 50. For these patients, surgery should be arranged as soon as possible to confirm the diagnosis so that chemotherapy can be started early if necessary. The results also revealed that the diagnostic specificity did not considerably decrease in the nonfunctional group (99.2%), was retained perfectly in the functional group (100%) and was higher than that of metanephrines and epinephrines (46). In other words, a score of less than 50 would almost always indicate PCC. Surgery is less urgent for the latter condition, since most PCCs are benign.

The current study had some limitations. First, this analysis was based on data from a single center, and the sample size was relatively small. However, owing to the low incidence of PAL and the application of bootstrapping and external validation, we still found our model to be helpful in clinical practice. However, additional studies and more profiles from other institutions or populations are warranted. Second, owing to the nature of the retrospective study, no PAL patients with unilateral lesions or with overt catecholamine-elevated lesions were included in the validation cohort. More data are needed to evaluate the efficacy of the nomogram in such patients. However, the results of our study did show concordance in the diagnostic specificity of the developed nomogram. In addition, we found the nomogram to be meaningful in clinical practice. Third, some variables were not included in our analysis due to data limitations, such as beta2-microglobulin, C-reactive protein, and mass density on CT imaging.

## Conclusions

In the present study, we incorporated independent determinant variables, including age, bilateral masses, lower HDL-C and higher LDH, into a nomogram to distinguish PAL from nonfunctional adrenal masses and functional PCC. This nomogram exhibited good discriminations. We found that the developed nomogram was capable of guiding the decision-making of adrenal incidentaloma and functional PCC.

## Data Availability Statement

The raw data supporting the conclusions of this article will be made available by the authors, without undue reservation.

## Ethics Statement

This study was approved by the Ethics Committee of West China Hospital of Sichuan University and adhered to the Declaration of Helsinki (No. 2019-556).

## Author Contributions

KY and YR designed the study. KY and FZ collected and cleaned the data. KY, FZ, and TC reviewed the medical records. KY and QPX analyzed the data and wrote the manuscript. QX helped in data collection. HT and YR supervised the study. All authors contributed to the article and approved the submitted version.

## Funding

This study was funded by the 1.3.5 Project for Disciplines of Excellence, West China Hospital, Sichuan University (ZYGD18022).

## Conflict of Interest

The authors declare that the research was conducted in the absence of any commercial or financial relationships that could be construed as a potential conflict of interest.
